# Three-Dimensional Digital Reconstruction of the Cerebellar Cortex: Lobule Thickness, Surface Area Measurements, and Layer Architecture

**DOI:** 10.1007/s12311-022-01390-8

**Published:** 2022-03-14

**Authors:** Junxiao Zheng, Qinzhu Yang, Nikos Makris, Kaibin Huang, Jianwen Liang, Chenfei Ye, Xiaxia Yu, Mu Tian, Ting Ma, Tian Mou, Wenlong Guo, Ron Kikinis, Yi Gao

**Affiliations:** 1 School of Biomedical Engineering, Health Science Center, Shenzhen University, Shenzhen, Guangdong, China; 2 Center for Morphometric Analysis, Departments of Psychiatry, Neurology, A. A. Martinos Center for Biomedical Imaging, Massachusetts General Hospital and Departments of Psychiatry and Radiology, Brigham and Women’s Hospital, Harvard Medical School, Boston, USA; 3 Department of Anatomy and Neurobiology, Boston University Medical School, Boston, USA; 4 Pengcheng Lab, Shenzhen, Guangdong, China; 5 Department of Electronic and Information Engineering, Harbin Institute of Technology Campus, Shenzhen, Guangdong, China; 6 Guangdong Provincial People’s Hospital, Guangdong Academy of Medical Sciences, Guangzhou, Guangdong, China; 7 Department of Radiology, Brigham and Women’s Hospital, Harvard Medical School, Boston, USA; 8 Marshall Laboratory of Biomedical Engineering, Shenzhen, Guangdong, China; 9 Shenzhen Key Laboratory of Precision Medicine for Hematological Malignancies, Shenzhen, Guangdong, China

**Keywords:** Cerebellum, Cerebellar cortical layers measurements, Surface area, Lobules, Laminar thickness measurements, Cerebellar cortical layers morphometry

## Abstract

The cerebellum is ontogenetically one of the first structures to develop in the central nervous system; nevertheless, it has been only recently reconsidered for its significant neurobiological, functional, and clinical relevance in humans. Thus, it has been a relatively under-studied compared to the cerebrum. Currently, non-invasive imaging modalities can barely reach the necessary resolution to unfold its entire, convoluted surface, while only histological analyses can reveal local information at the micrometer scale. Herein, we used the BigBrain dataset to generate area and point-wise thickness measurements for all layers of the cerebellar cortex and for each lobule in particular. We found that the overall surface area of the cerebellar granular layer (including Purkinje cells) was 1,732 cm^2^ and the molecular layer was 1,945 cm^2^. The average thickness of the granular layer is 0.88 mm (±0.83) and that of the molecular layer is 0.32 mm (±0.08). The cerebellum (both granular and molecular layers) is thicker at the depth of the sulci and thinner at the crowns of the gyri. Globally, the granular layer is thicker in the lateral-posterior-inferior region than the medial-superior regions. The characterization of individual layers in the cerebellum achieved herein represents a stepping-stone for investigations interrelating structural and functional connectivity with cerebellar architectonics using neuroimaging, which is a matter of considerable relevance in basic and clinical neuroscience. Furthermore, these data provide templates for the construction of cerebellar topographic maps and the precise localization of structural and functional alterations in diseases affecting the cerebellum.

## Introduction

The cerebrum is an essential component of the brain that integrates sensory information to coordinate voluntary actions. Given the importance of this structure in all vertebrates, researchers have made great efforts to resolve its detailed structure. As a result, we now know the average surface area of various cerebrum regions [1, 2], as well as the cortical [3] and laminar thicknesses [4] of the gray matter (GM) and the cortical folding patterns [5, 6]. We have even generated functional maps of the cerebrum [7, 8].

**Fig. 1:**
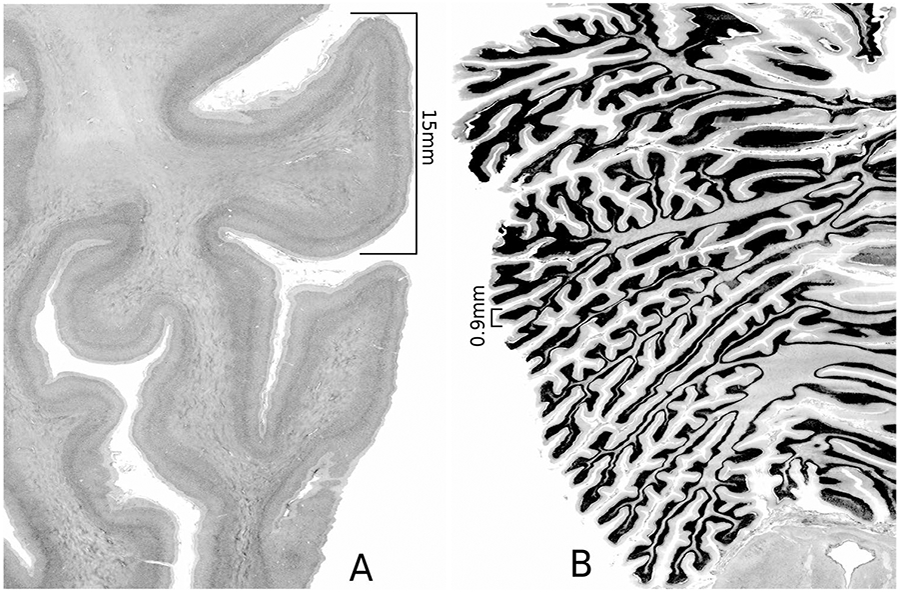
A comparison of the cerebral gyrus and the cerebellar lobules under the same scale. **A**. Cerebral folding. **B**. Cerebellum folding. The width of a typical fold in the cerebrum is 15 mm, while that of a typical fold in the cerebellum (a folium) is 0.9 mm

By contrast, we know relatively less about the cerebellum, despite it being the second largest organ of the brain located below to the occipital lobes of the cerebrum and behind the brainstem. To date, only a small number of preliminary structural [9, 10] or functional [11-14] studies have been performed, which have been largely limited by the resolution of available imaging devices. It is well known that the cerebellum exhibits a fine folding pattern, which is quite different compared to that of the cerebrum (see Fig. 1 for a comparison). Cerebellar cortical folds extend into groups of horizontal and parallel grooves, and these folds contain further, smaller folds that further extend its surface area. In anatomical terms, its accordionlike appearance reflects its high degree of folding, which increases considerably its surface area. Deeper fissures divide morphologically the cerebellum into lobes and lobules, whereas more swallow fissures incise the banks of the deeper fissures, mostly in the transverse orientation, to generate the cerebellar cortical ridges called folia [15]. Underlying the cerebellar cortex is white matter made-up of incoming and outgoing axons, which constitute the principal wiring mass of cerebellar extrinsic connectivity [15-18]. Given the spatial resolution of the cerebellum even at a gross level of morphological description, quantitative analyses deriving cortical thickness, surface or volume, are more arduous than in the cerebrum [19, 20]. Furthermore, as shown in Fig. 1, this complex structural blueprint of orthogonally oriented deep and shallow fissures, which provides the cerebellum with a large surface area, is an additional factor in rendering the studies of its cortical surface area and thickness a much more challenging task, compared with the cerebral cortex.

Histologically, the cerebellar cortex comprises three cellular layers, namely, a deeper granular cell layer, the Purkinje cell layer, and the most superficial molecular layer [21]. The granular layer is the thickest and is composed of small and numerous granular cells. The Purkinje layer contains only a layer of Purkinje cells while the outer-most molecular layer, which consists mostly of interneurons, is made up of the flattened dendrites of the Purkinje cells connected to each other by a vast array of parallel fibers. When compared the cerebral gyrus and the cerebellar lobules under the same scale (Fig. 1), the width of a typical fold in the cerebrum is about 15 times that of a typical fold in the cerebellum (a folium).

Recently, Kalanjati et al. [22] measured the molecular layer thickness of local cerebellar slices. Specifically they isolated and stained slices of the cerebellum, which they analyzed at the microscopic level. Spatial resolution and tissue contrast have been serious barriers in studying the cerebellum in fine-grained structural detail using magnetic resonance imaging (MRI), especially in vivo. In fact, even using a very high magnetic field, the layers of the cerebellar cortex and the gaps between the cerebellar cortical folds can not be clearly observed in the MR images produced. Thus, to overcome this critical obstacle, most of the detailed structural analysis has been performed using human ex vivo material. This was pioneered by seminal studies in the early 2000s [23]. Recently, Sereno et al. [24] scanned a whole, postmortem cerebellum at 9.4 T and reconstructed the surface of the cerebellum down to the level of individual folia. They found that the total shrinkage-corrected surface area (1,590 cm^2^) was larger than expected, constituting 78% of the total human neocortex surface area. Even though this study calculated the cerebellar surface area down to the level of individual folia, 9.4 T MRI is not sufficient to study the layered structures or the thickness of the cerebellum.

A point-wise thickness distribution of the entire cerebellum remains an unstudied problem. Moreover, how the fine-scale surface area of the cerebellum compares with the cerebrum, particularly in the same subject, remains unclear. Addressing these knowledge gaps is crucial given the importance of this structure. Furthermore, enabling the morphometric characterization and quantitative assessment in thickness of individual cellular layers opens up a window in structural studies of cerebellar connectivity at a level that, to our knowledge, has not been achieved to date in vivo in humans or in the non-human primate. Early studies predominantly associated the cerebellum with motor functions; however, more recent studies have shown that the human cerebellum is also associated with non-motor functions [25] including emotion [26] and language cognitive functions such as language [27] and memory [23, 28-32]. Importantly, in the clinical domain, several disorders such as epilepsy [33], schizophrenia [34], multiple system atrophy [35], and autism [36] have been associated with deficits at the level of the cerebellum. Given that cortical thickness is a very robust brain parameter and relatively invariant during mammalian evolution [37-39], it is imaginable that the cerebellar cortex thickness could well be a useful nervous tissue correlate in studying the development and aging of brain tissue [40] and how cerebellar cortical thickness changes and of its individual cellular layers may be associated with neurological and mental decline as well as with other neuropsychiatric diseases [25].

BigBrain is a 3D histological model of the human brain at 20-μm isotropic resolution. Herein, we aimed to produce a map showing the thicknesses and surface areas of the different cerebellar layers at 20-μm resolution, using data from the BigBrain project [41].

## Materials and Methods

### Data Collection and Preprocessing

The BigBrain project involved the volumetric reconstruction of histological scans made of a postmortem brain, taken from a 65-year-old male who died without any neurological or psychiatric diseases in clinical records [41]. The dataset consists of serially sectioned 2D sections of silver-stained brain tissue slice of 20 μm thick. Each slice is then imaged with digital microscope at a resolution of 20 × 20 μm per pixel. We reconstruct a 3D volume using the original BigBrain slices forming a 20 × 20 × 20 μm per pixel resolution volume. All the subsequent segmentation and measurement are based on this high-resolution volume. The data were downloaded from the BigBrain website (ftp://bigbrain.loris.ca.).

We performed all segmentations at 20 × 20 × 20 μm resolution, and the single file containing only the cerebellum region was 62 GB.

### Laminar Segmentation

The laminar structure of the cerebellum, namely, the granular cell layer (including the single cell Purkinje cell layer) and the molecular layer, was segmented using the semiautomatic software 3D Slicer [42] and a 2D convolutional neural network (CNN) based approach [43].

**Fig. 2:**
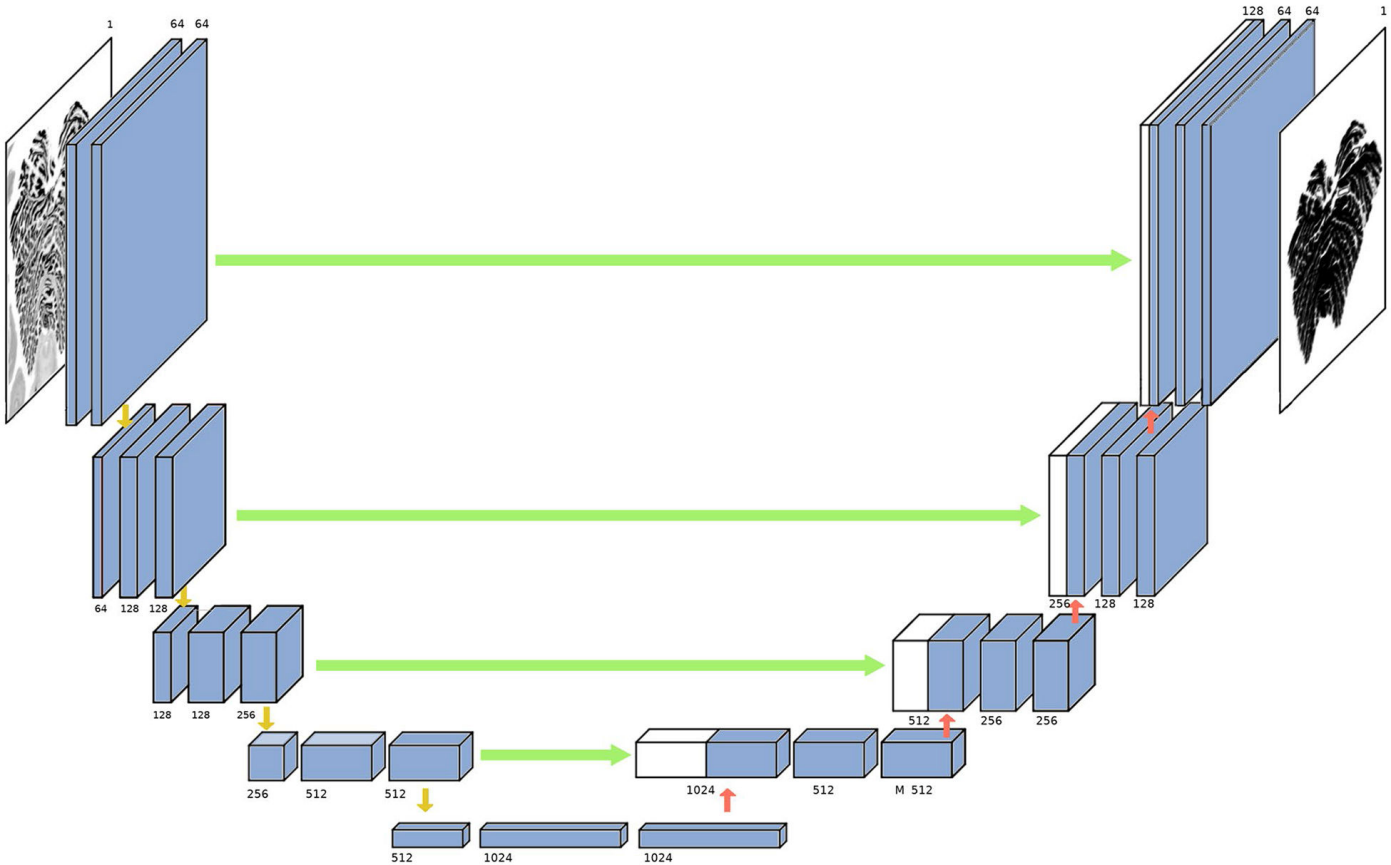
The U-net architecture. The U-net architecture comprised convolutional encoding and decoding units that took the cerebellar image as the input and produced the molecular layer surface map

The Purkinje cells layer contains a monolayer of Purkinje cells, which can only be observed at high magnification histological scantion [22]. Granular cell and Purkinje cell are stained darker than cell of molecular layer [41]. Thus, thickness of granular layer includes the single cell Purkinje cell layer. First, manual segmentation was performed on four axial slices: one near the top, one near the base, and the two near the middle of the volume. These four slices served as a training set for the patch-based (patch size 256 × 256) CNN. Once the training converged, the model was applied to the remaining 2,366 slices to produce the volumetric segmentation of the entire cerebellum (Fig. 2). The manually corrected segmentation results were verified by a neuroanatomist with more than 10 years of experience.

The segmentation was performed using the semiautomatic algorithms/software (Grow From Seed in 3D Slicer [42]), and the results have been validated by a senior neuroanatomist. Using the BigBrain dataset, we first segmented the cerebellum into the cerebellar white matter, the granular and molecular layers. We then computed the subsequent surface areas and thicknesses for each segment.

### Lobule Segmentation

An interactive segmentation module (Fast Grow Cut) in 3D Slicer [42] was used to segment each cerebellar lobule. Then, manual segmentation was performed, as previously described [44]. The lobule segmentation results were also verified by our neuroanatomist.

### Data Shrinkage Correction

The entire BigBrain sample had a volume of 1,216 cm^3^. However, after preparation, the volume reduced to 682 cm^3^, resulting in a 3D volumetric shrinkage factor of 1.931 or an isotropic length-based (1D) shrinkage factor of 1.245(1.245^3^ = 1.931) [4]. Shrinkage compensation was therefore necessary to analyze the thickness of the cerebellar cortex. Thus, all measurements reported herein were corrected for by this factor.

### Thickness Measurements

Following volumetric layer segmentation, the thicknesses of the granular and molecular layers were calculated. Specifically, for each point on the interior surface of the two layers, the closest point on the outer surface was identified, and the distance between them defined the local thickness. These calculations were carried out in three dimensions, in contrast to previous histology slice-based cerebellar thickness calculations, which have been performed in two dimensions [22]. Due to the possible variations in slice orientation, the two-dimensional thickness will always be greater or equal to the three-dimensional thickness. Inevitably, this process induces bias in the estimation of the thickness across the entire cerebellum. As a result, the three-dimensional thickness computed herein should provide a more accurate point-wise measurement and global variation estimation.

To determine the thickness distribution of the cortical layers, the influence of a small number of too large or too small data was removed by fitting a gamma distribution on the granular layer data for each lobule. Conversely, a Gaussian mixture model was fitted on the molecular layer data for each lobule. Furthermore, the eroded parts of the molecular layer were removed and the Gaussian model was kept for most of the results.

### Area Measurement

Once segmented, a triangulated mesh was generated using a marching cube algorithm [45], and the surface area was computed on the mesh.

## Results

### Cerebellar Segmentation

We first determined whether the laminar thickness of the whole cerebellum is consistent with the previous local histology-based measurements.

As mentioned in the above “Materials and Methods” section, during the sample preparation stage, the BigBrain sample shrank significantly. The linear shrinkage factor was estimated to be 1.245 [4]. We thus corrected all values for this shrinkage factor.

### Cerebellar Surface Area

**Fig. 3:**
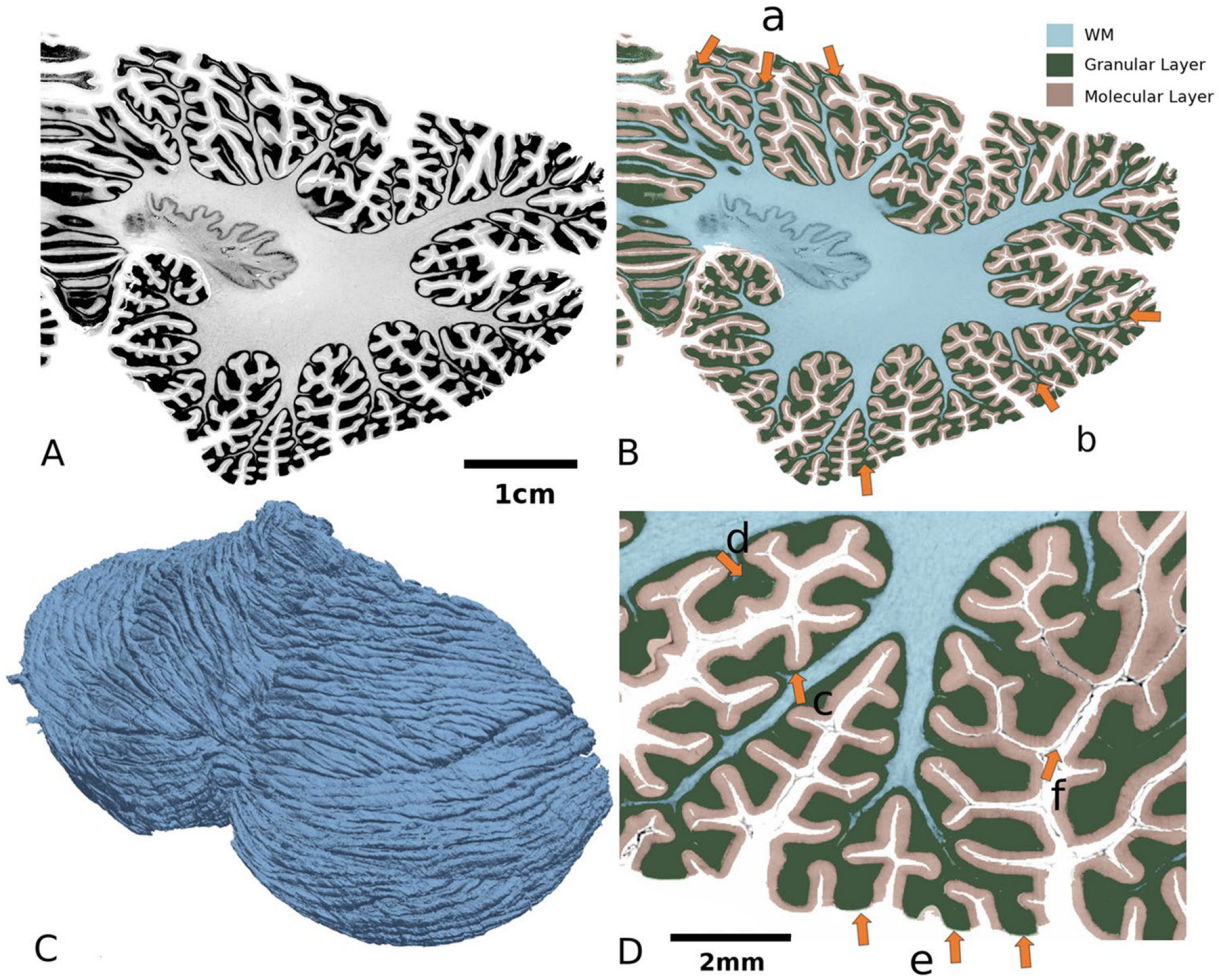
Segmentation of cerebellar layers. The white matter (WM) and granular layer were segmented by an interactive method [42] and the pial surface was segmented by automatic deep learning-based method [43], see “Materials and Methods” section for detail. **A**. Coronal, high-resolution histological sections of the BigBrain cerebellum. **B**. Segmentation of the cerebellum shown in one exemplar slice. The WM penetrates into each folium (arrow a), whereas the WM in the outer folia disappears (arrow b). **C**. A three-dimensional reconstruction of the BigBrain cerebellar surface. This mesh contains 159,923,340 vertexes and 319,946,592 triangles. **D**. Local variations in cerebellar thickness. The granular layer is thicker at the crown of the gyrus (arrow d) and thinner in the depth of a sulcus (arrow c). The molecular layer is lost in certain out-facing regions (arrow e), while the folded surface remains complete (arrow f), which induces some thickness calculation artifacts

We next calculated the total pial surface area of the BigBrain cerebellar cortex. The surface area was 1,945 cm^2^ (Fig. 3C), which is, to the best of our knowledge, considerably larger than any reported estimate [4, 46-48] to date. By contrast, the BigBrain cerebral cortex area (after correction for the shrinkage) was 2864 cm^2^ (= 1,848 × 1.245^2^). (It is noted that the cerebral surface area of the BigBrain reported in the supplemental material of [4] was 3050 cm^2^. However, such a number was calculated after registering the BigBrain cerebral surface to that of a group of ADNI [49] MRI volumes. As a result, the two numbers, 2864 and 3050, are not directly comparable.) Therefore, the pial surface ratio between the cerebellum and the cerebrum is 68% (1945/2864). While this ratio is smaller than previous estimation, those ratios have been computed based on data derived from different subjects [50] than the BigBrain data analyzed herein. To the best of our knowledge, this is the first intra-subject cerebellar-cerebral area comparison at level of cerebellar folia.

### Cerebellar Laminar Thickness (i.e., Thickness of Cerebellar Cortical Layers)

Subsequently, we studied the average cerebellar thickness; the thickness of the BigBrain across all layers was 1.2 mm. It should be noted that while this finding is consistent with data derived from local histological analyses [21], histology-based approaches have typically been unable to map the laminar thickness across the entire cerebellum.

**Fig. 4:**
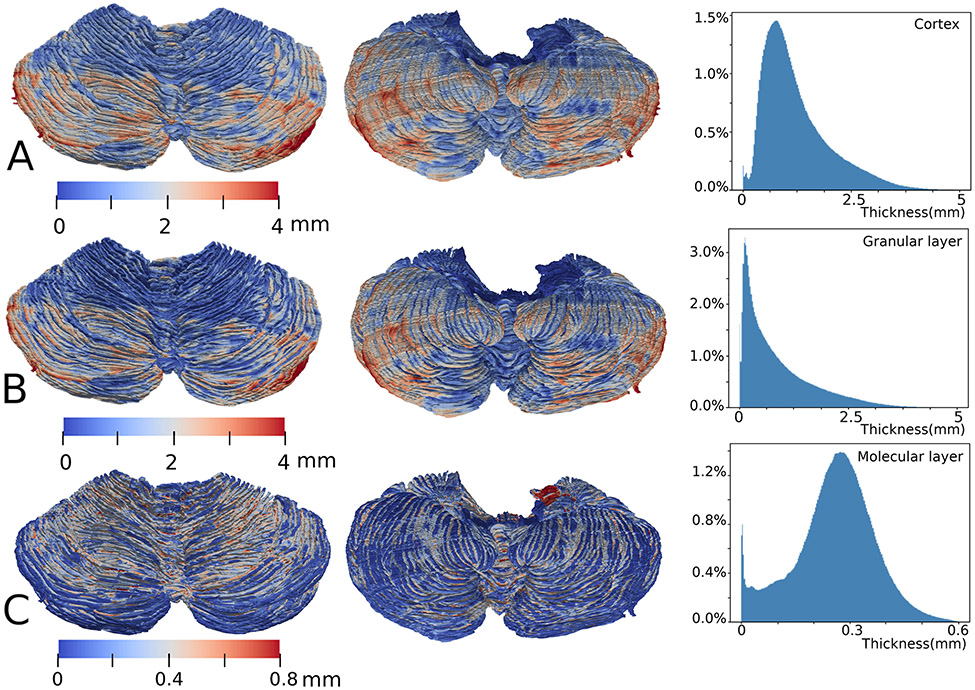
The thickness of the cerebellar cortex and cortical layers. In all the three rows, downward points to the posterior or caudal of the subject. **A**. A superior (left) and inferior (middle) view of the cerebellar cortical thickness mapped onto the pial surface and a histogram of the cortical thickness distribution (right) are depicted. **B**. The thickness of the granular layer (including the Purkinje cell layer) and a histogram of the thickness distribution are shown. **C**. The thickness of the molecular layer mapped onto the Purkinje cell surface and a histogram of the thickness distribution are illustrated. The spike on the histogram is close to thickness of 0 mm and corresponds to the dark blue-colored gyri on the inferior side on the left and in the middle. The dark blue-colored regions are due to the absence of the molecular layer in the original BigBrain data, possibly due to tissue damage during sample processing

Next, we assessed the laminar thicknesses of the different cerebellar layers (Fig. 4). We found that the granular layer contributes the most (73%) to the overall cerebellar thickness. It needs to be specified that, because the Purkinje layer is only single cell thick, we did not considered this layer as a distinct region of interest (ROI) in our thickness computations and was included in the ROI of the granular layer.

While the curvature of the cerebral cortex is mostly small, the cerebellar surface makes frequent, sharp turns. This phenomenon is most prominent in the granular layer. For example, the local sharp turn of the granular layer results in a notable thickening (7.5 times) of this high curvature region (Fig. 3D).

The highly curved nature of the cerebellar surface results in prominent variations in local cerebellar thickness, ranging from 0.2 to 2 mm. By contrast, the thickness of the cerebral cortex varies at a larger scale. That is, the cerebral thickness is locally more uniform but varies significantly from one cortical region to another [4]. Specifically, we found that in 95% of the cerebellar surface, the range of the molecular layer thickness was between 0.1 and 0.5 mm (mode = 0.27 mm).

The thickness of the molecular layer seems to follow a Gaussian distribution, while that of the granular layer follows an entropy maximizing gamma distribution, which spans a much wider range. This finding might be because this layer is much thicker at the positive-curvature folia region (typical granular thickness at the region pointed by Fig. 3 arrow d = 0.9 mm) than the negative-curvature concave region (typical granular thickness at the region pointed by Fig. 3 arrow c = 0.11 mm). Ninety-five percent of the thickness values range from 0.02 to 3.5 mm. Moreover, the granular layer increases in thickness from superior to inferior.

### Lobule Surface Area and Thickness

Subsequently, we analyzed each lobule by dividing the Big-Brain cerebellum into lobules I to X [51-53].

**Fig. 5:**
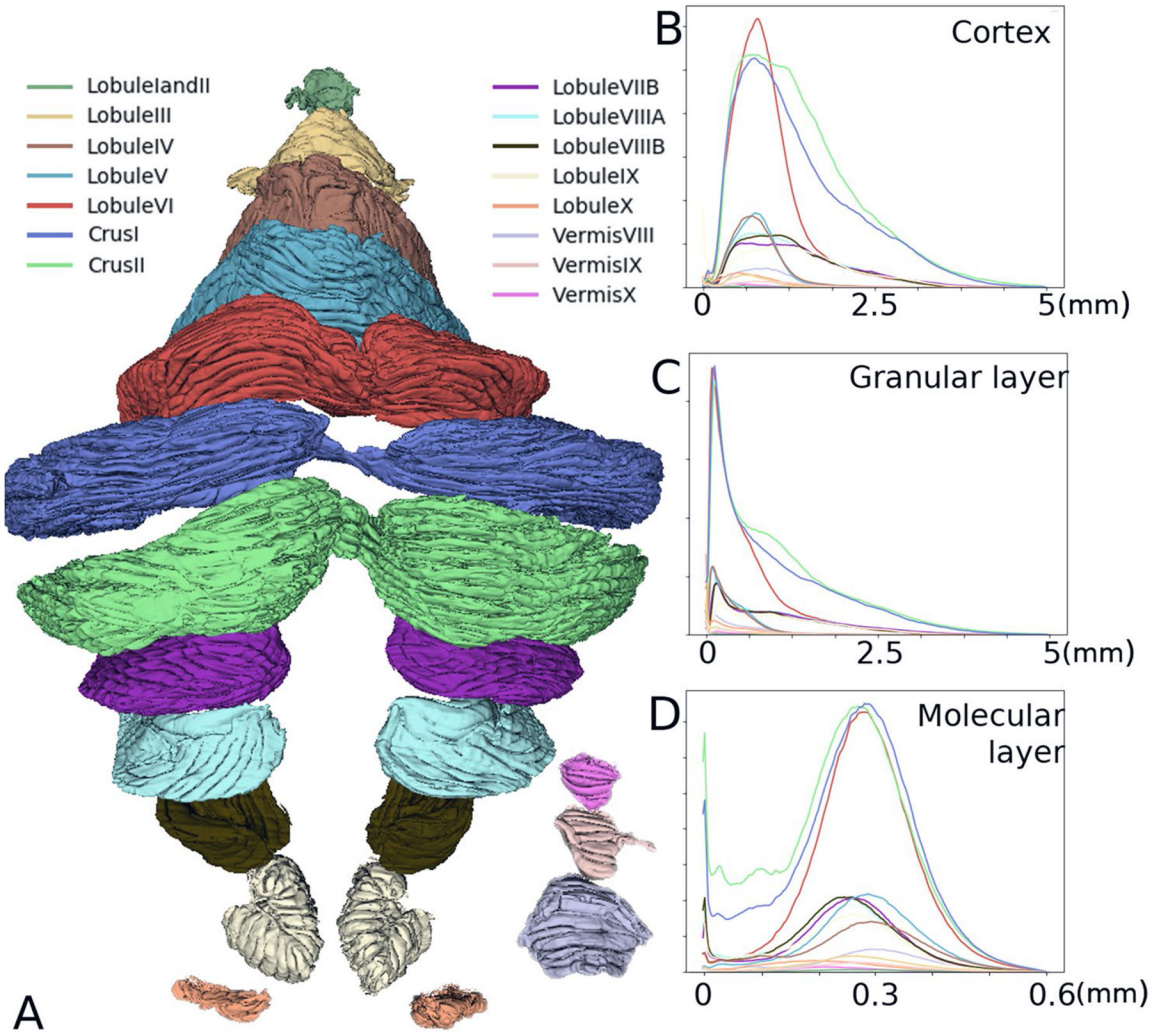
Segmentation and corresponding thicknesses of the cerebellar lobules. **A**. Segmentation of each lobule down to the individual folia resolution. **B-D**. Thickness histograms of the cerebellar cortex (**B**), granular (**C**), and molecular (**D**) layers of each lobule

We observed that the granular layer thickness significantly changes in the posterior cerebellum compared to the anterior cerebellum (Fig. 5 and Table 1). Specifically, the thickness of > 90% of the granular layers of lobules I–VI, X and the vermis VIII–X is 0–1 mm. By contrast, lobules VII to IX are thicker with values in the range of 0–3 mm.

### Molecular Layer Thickness

**Fig. 6:**
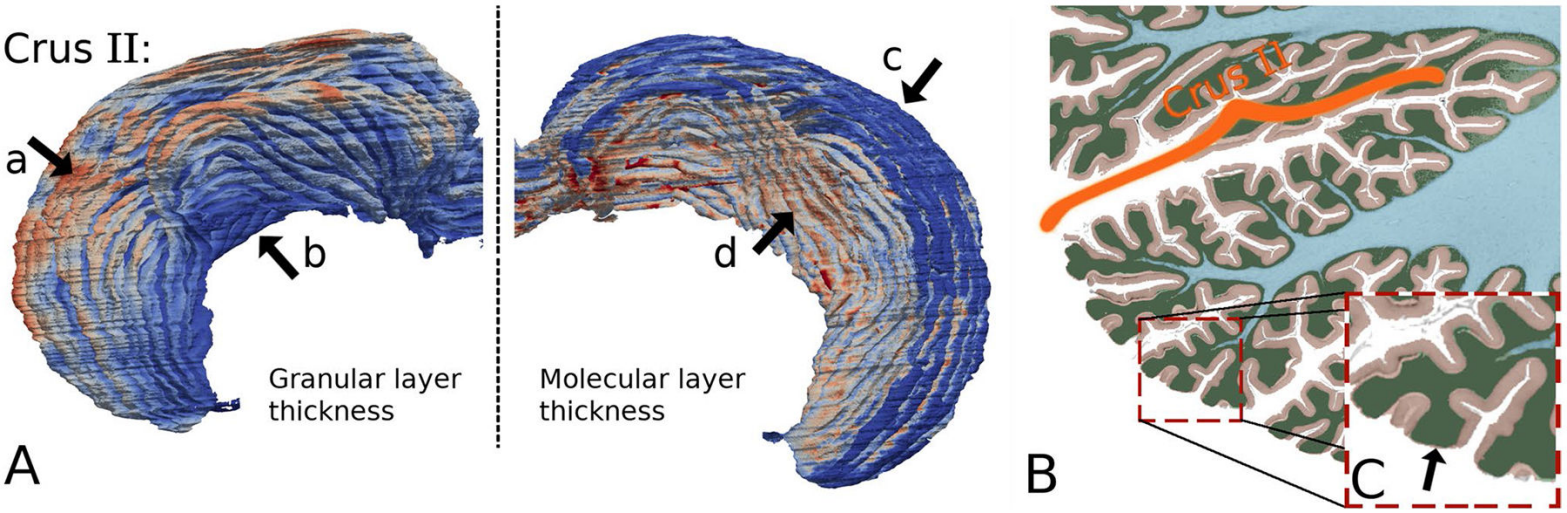
Three-dimensional image of a single lobule and its partial display. **A**. Thickness maps of the granular (left) and molecular layers (right) of lobule crus II. Arrow a indicates the tip of the folia, where the granular layer is thick, while arrow c indicates where the molecular layer is very thin. **B-C**. A cross-sectional view of crus II B and the magnified sub-region **C**. The apparent thickening of the granular layer might be due to the depth of the white matter. The thinning of the molecular layer might be due to erosion during sample preparation

During our analyses, we noted that a considerable portion of the molecular layers of crus I and crus II have thickness close to 0 (Fig. 5D, the dark-blue gyri in Fig. 4B, and Fig. 6A arrow c). We thus analyzed more closely a cross-sectional view of crus II (Fig. 6B-C) and saw that in the out-facing gyrus regions (at the tip of the folia), the molecular regions are in fact worn out. These regions of abrasion contributed to the close-to-zero thickness (Fig. 4C). We believe that such a phenomenon could be due to problems in the sample preparation as these regions are completely exposed.

**Fig. 7:**
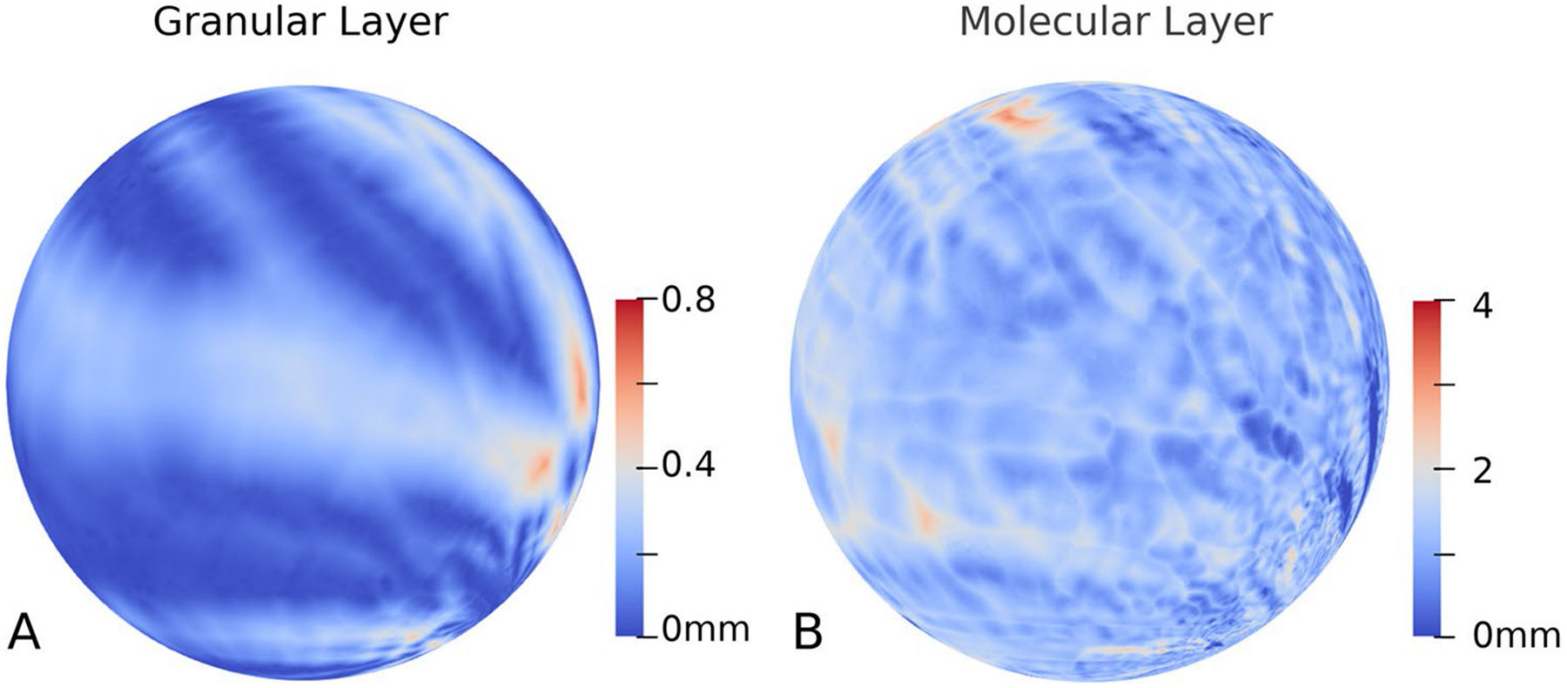
The local surface with thickness defined on them using the conformal spherical parameterization. **A**. Conformal mapping of the granular surface to the sphere. **B**. Conformal mapping of the molecular layer surface to the sphere

Inflating the surface to better visualize the distance map on the grooved-in regions would be ideal to determine the cerebellar gyri. Unfortunately, given the very large mesh size (~ 160 M vertexes, which is 35 times higher of that previously reported in the postmortem MRI-based study [24]), currently available inflation software, such as the FreeSurfer [54], is unable to handle such a fine-scale mesh. Therefore, in order to get the inflated view, we inflated the local surface with thickness defined on them using the conformal spherical parameterization. To this end, we used the itkConformalFlattening filter in the InsightToolkit [55, 56]. Figure 7 shows the spherical parameterized mesh with the thickness map used as texture mapping. As can be clearly observed, the stripe pattern on the sphere corresponds to the sulci and gyri of cerebellum. And the thickness variation on the granular layer is quite significant. On the contrary, the thickness distribution of molecular layer was relatively uniform: there was no significant difference between the thickness of the sulci and gyri.

In contrast to the crus I and crus II regions, the superior regions of the cerebellum, roughly corresponding to lobule V, showed intact molecular layers. These regions are located the occipital lobe of the cerebrum and are thus protected from erosion.

### Granular Layer Thickness

The granular layer was found to be very thick at the tip of the folia (Fig. 6A, left). Indeed, as the thickness of the granular layer is computed as the distance between the outer surface of the granular layer and that of the WM, the thickness is markedly affected by the penetration of the white matter. The imaging resolution limits the analysis, and thus, the thin white matter fiber seen in the BigBrain data (Fig. 6C) only penetrates half-way into the folia shown herein. Thus, the thickness of the granular layer at the tip of the folia might be over-estimated. It should be pointed out that without finer and more sensitive imaging technology, we cannot conclude whether this measurement is caused by the absence of WM itself, or by the insufficiency of the imaging resolution.

### Other Observations

When examining the detailed geometry of the lobules and folia, we observed that the crest of a lobule sometimes disappeared when it approached the midline, and then reappeared in the fissure of opposite hemisphere (Fig. 6). This is consistent with a previous observation [24]. Folds at the fundus of the fissure sometimes spanned across two lobules and the folia relative topography varied with respect to the long axis of a lobule. These issues affect the precise segmentation of the lobules at high resolution. Interestingly, however, the surface area of lobule VII together with lobule VIII was found to be twice the total area of lobules I to VI (Table 1). This measurement exceeded previously reported estimates [24, 57].

## Discussion

In the present study, we were able to measure the total cortical thickness of the BigBrain cerebellum and also the thicknesses of the granular and molecular layers specifically to explore the histological drivers of MRI-based thickness gradients. Overall, the pattern of thickness in the BigBrain cerebellum is consistent with local histological studies of cortical thickness, such as that of Braitenberg and Atwood [21]. By segmenting the high-resolution BigBrain cerebellar cortex, we measured the thickness of the cortical layers of the entire cerebellum in three dimensions. Our measurements were not only consistent with those derived from previous local histological studies, but also revealed new, global variations in thickness. A notable finding is that the total surface area of the cerebellum is larger than previous estimates made based on postmortem MRI studies. However, the cerebellar-to-cerebral area ratio is smaller than previously described [24], which was the largest ratio reported to date. Furthermore, we assessed quantitatively the laminar characteristics of the cerebellar cortex.

Our observations indicated that the thickness of the granular layer in the posterior cerebellar lobe, i.e., lobules VI–VIII, associated with cognitive functions was considerably higher as compared to that of the other lobes. Specifically, the average thickness of the granular layer in lobules VI to VIII was 1 mm, whereas that of lobules I to V, which are associated with balance, posture, and sensorimotor functions, was 0.3 mm and that of lobules IX and X, oculomotor, which are related to autonomic and emotional functions, was 0.875 mm (Table 1). Given the tremendous increase in cognitive capacities of the human brain in evolutionary terms, it seems plausible that cerebellar regions such as crus I and crus II have expanded in thickness to serve a high demand in cognitive functioning. Although this has been indicated in early volumetric studies for the entire crus I and crus II regions [20], herein is demonstrated the relevance of the granular layer in particular. Given the architecture of this layer in the wiring diagram of the cerebellum, this finding has critical implications with respect the structural and functional connectivity of the cerebellum. Furthermore, these observations can be translated in the domain of neuroimaging with imaginable implications for clinical studies.

It is conceivable that the capability to determine quantitatively the laminar architecture of the cerebellum will have significant implications for studying cerebellar structural cellular architecture in vivo. This level of histological characterization is not limited to the cellular level of investigation but is directly associated with the issue of understanding and elucidating the intrinsic connectional architecture of the cerebellum, which is of high neurobiological and clinical relevance. Furthermore, this pioneering investigation will open up the query for novel methodological strategies leading to deeper insight regarding the extrinsic connectivity of this complex structure and, thus, of its structural and functional organization within the central nervous system. Moreover, these data provide for the first time, digitized templates of cerebellar computational anatomy for the construction of topographic maps of this structure including a laminar level of characterization. This information can be used for anatomical localization of lesions and function as well as for the precise morphometric definition of cerebellar regions of interest [19, 20, 24] and of lesions in cerebellar-related diseases [30, 31, 51, 58-60].

The information on laminar architecture provided herein in this manuscript has immediate applicability in computational neuroanatomy using imaging technology. The level of histological representation depicted in this analysis has the potential to make this paper an important stepping-stone to enable the determination of the laminar origins and terminations of specific connections of the cerebellum using MRI. This level of detail regarding cellular layers is essential for interrelating connectional patterns with intrinsic and extrinsic cerebellar architectonics [61]. Similar scenarios have been implemented in the past with successful results. A clear example of how sophisticated anatomical knowledge can be transferred from the field of traditional neuroanatomy to the field of computational neuroanatomy is the following. In the early 2000s, a thickness measurement of superficial cerebral white matter (SWM), including U-fibers, was adopted in MRI-based morphometric analysis, based on purely histological observations and measurements of Nissl and myelin-stained serial sections of two human brains [62-64]. This information was eventually translated into algorithmic development for the FreeSurfer segmentation and cerebral cortical parcellation for automated analysis [65, 66]. Likewise, the histology-based computational digital reconstruction information provided in this study could be transferred to neuroimaging of the cerebellum, given the available state-of-the-art technology as reported recently by Sereno and colleagues [24]. Thus, this study can be viewed as a significant incremental step toward elucidating connectional neuroanatomy and integrating it with morphometry of MRI cerebellar computational neuroanatomy. The immediate applicability of this knowledge is imaginable in neural systems neuroscience and brain organization. This is because cerebellar organization and integration within the central nervous system is tightly related to its anatomical connections, which are architectonically specific, relating the cerebellum to the cerebrum, brainstem, and spinal cord via distinct neural systems that follow precise and specific laminar origins and terminations [29, 32, 67]. Therefore, the capability to segment differentially the granular cell layer allows the determination of axonal terminals reaching from the pons, brainstem, spinal cord, and cerebellar nuclei en route to Purkinje cells of the cerebellum. Moreover, by differentiating the granular cell layer from the overlying Purkinje cell layer and the molecular layer, the cerebellar output circuitry can be determined, given that the origins of the efferent axons are almost entirely from Purkinje cells and to a lesser extent from the cerebellar nuclei [15-18]. This detailed definition of cerebellar structural connectivity can be used as a basis for quantitative morphometric analysis in neuroanatomical and clinical studies as well as for functional mapping of the cerebellum in cognitive and systems neuroscience. It has been conceived from a neural systems perspective based on current knowledge of cerebellar histology, anatomic connectivity, functional correlations, and clinical and behavioral manifestations in the human and nonhuman primate and can be fully integrated into current neuroimaging of the cerebellum [20, 24, 58-60].

Furthermore, the high-resolution surface of individual cerebellar cortical layers of individual folia is now publicly available. Thus, the complete cerebellar cortical layer surface can also serve as a map of functional mapping for the cerebellar cortex.

## Limitations

Because all the measurements were made based on data from a single subject, we cannot rule out that the patterns we observed here are unique to this subject. Furthermore, torn patches of zero thickness vertices in the molecular layer were removed and were not included in the statistical analyses. Future work is necessary to establish the inter-subject and age-dependent variability in laminar structure of the cerebellar cortex. Another limitation is that numerous molecular regions were eroded or degraded, which generated some areas with an apparent zero thickness introducing possible calculation artifacts.

## Figures and Tables

**Table 1:** Surface area, GM volume, and thickness of each cerebellar lobule

Lobule	Area (mm^2^)	GM volume (mm^3^)	Thickness (means (mm), std)	
			Granular layer	Molecular layer
Lobules I, II	230	95	0.13 ± 0.18	0.36 ± 0.11
Lobule III	1425	617	0.29 ± 0.31	0.23 ± 0.10
Lobule IV	6101	2333.7	0.41 ± 0.35	0.34 ± 0.09
Lobule V	6986	2919.68	0.40 ± 0.30	0.34 ± 0.08
Lobule VI	20,551	8476	0.61 ± 0.54	0.33 ± 0.08
Crus I	27,801	11,253	1.04 ± 0.95	0.32 ± 0.08
Crus II	32,611	12,608	1.09 ± 0.96	0.31 ± 0.08
Lobule VIIB	6016	2742	1.11 ± 0.93	0.30 ± 0.08
Lobule VIIIA	6732	2959	1.03 ± 0.85	0.30 ± 0.08
Lobule VIIIB	6630	2890	1.01 ± 0.84	0.30 ± 0.09
Lobule IX	5806	2401	1.08 ± 1.09	0.30 ± 0.08
Lobule X	1079	359	0.67 ± 1.04	0.17 ± 0.08
Vermis VIII	2262	996	0.53 ± 0.41	0.35 ± 0.08
Vermis IX	840	417	0.67 ± 0.62	0.33 ± 0.09
Vermis X	433	140	0.35 ± 0.37	0.27 ± 0.09
Total	125,576	51,252	0.88 ± 0.84	0.32 ± 0.08

Abbreviation: *GM*, gray matter

## Data Availability

The segmented surface results for the granular and molecular layers will be publicly available at 106.52.156.235. Gamma distribution of granular layer lobules: 106.52.156.235/Data1.xlsx Gaussian distribution of molecular layer lobules: 106.52.156.235/Data2.xlsx Surface mesh of granular layer surface and each lobule: 106.52.156.235/GranularLayerMeshAndLobules.vtp Surface mesh of molecular layer surface: 106.52.156.235/MolecularLayerMesh.vtp Since the very fine-scale meshes are of huge size (9 GB), the data are uploaded to the personal website above during review process. If required, we are agreeable to upload them to the designated server.
