# Correction: Three-Dimensional Digital Reconstruction of the Cerebellar Cortex: Lobule Thickness, Surface Area Measurements, and Layer Architecture

**DOI:** 10.1007/s12311-023-01603-8

**Published:** 2023-09-05

**Authors:** Junxiao Zheng, Qinzhu Yang, Nikos Makris, Kaibin Huang, Jianwen Liang, Chenfei Ye, Xiaxia Yu, Mu Tian, Ting Ma, Tian Mou, Wenlong Guo, Ron Kikinis, Yi Gao

**Affiliations:** 1 School of Biomedical Engineering, Health Science Center, Shenzhen University, Shenzhen, Guangdong China; 2 Center for Morphometric Analysis, Departments of Psychiatry, Neurology, A. A. Martinos Center for Biomedical Imaging, Massachusetts General Hospital and Departments of Psychiatry and Radiology, Brigham and Women’s Hospital, Harvard Medical School, Boston, USA; 3 Department of Anatomy and Neurobiology, Boston University Medical School, Boston, USA; 4 Pengcheng Lab, Shenzhen, Guangdong China; 5 Department of Electronic and Information Engineering, Harbin Institute of Technology Campus, Shenzhen, Guangdong China; 6 Guangdong Provincial People’s Hospital, Guangdong Academy of Medical Sciences, Guangzhou, Guangdong China; 7 Department of Radiology, Brigham and Women’s Hospital, Harvard Medical School, Boston, USA; 8 Marshall Laboratory of Biomedical Engineering, Shenzhen, Guangdong China; 9 Shenzhen Key Laboratory of Precision Medicine for Hematological Malignancies, Shenzhen, Guangdong China

**Correction to: The Cerebellum (2022) 22:249-260**

10.1007/s12311-022-01390-8

We have updated the url for data access to google drive at: https://drive.google.com/drive/folders/16pho-in8xEZFRxI9Vbl0KMhvqzStt56A, for its better and more flexible download/upload functions.

For further questions or require any assistance, please feel free to reach out with the authors.

